# Effectiveness of Eriomin® in managing hyperglycemia and reversal of prediabetes condition: A double‐blind, randomized, controlled study

**DOI:** 10.1002/ptr.6386

**Published:** 2019-06-11

**Authors:** Carolina B. Ribeiro, Fernanda M. Ramos, John A. Manthey, Thais B. Cesar

**Affiliations:** ^1^ Department of Food and Nutrition, School of Pharmaceutical Sciences Sao Paulo State University—UNESP Araraquara São Paulo Brazil; ^2^ U.S. Horticultural Research Laboratory Agricultural Research Service, USDA Port Fierce Florida

**Keywords:** flavonoids, hyperglycemia, insulin resistance, prediabetic state

## Abstract

This study evaluated the potential effectiveness of different doses of Eriomin® on hyperglycemia and insulin resistance associated with other metabolic biomarkers in prediabetic individuals. Prediabetes patients (*n* = 103, 49 ± 10 years) were randomly divided into four parallel groups: (a) Placebo; (b) Eriomin 200 mg; (c) Eriomin 400 mg; and (d) Eriomin 800 mg. Assessment of biochemical, metabolic, inflammatory, hepatic, renal, anthropometric markers, blood pressure, and dietary parameters were performed during 12 weeks of intervention. Treatment with all doses of Eriomin (200, 400, and 800 mg) had similar effects and altered significantly the following variables: blood glucose (−5%), insulin resistance (−7%), glucose intolerance (−7%), glycated hemoglobin (−2%), glucagon (−6.5%), C‐peptide (−5%), hsCRP (−12%), interleukin‐6 (−13%), TNFα (−11%), lipid peroxidation (−17%), systolic blood pressure (−8%), GLP‐1 (+15%), adiponectin (+19%), and antioxidant capacity (+6%). Eriomin or placebo did not influence the anthropometric and dietary variables. Short‐term intervention with Eriomin, at doses of 200, 400, or 800 mg/day, benefited glycemic control, reduced systemic inflammation and oxidative stress, and reversed the prediabetic condition in 24% of the evaluated patients.

**Abbreviations**
ABTS: 2,2′‐azino‐bis (3‐ethylbenzothiazoline‐6‐sulphonic acid)
ADA: American Diabetes Association
ALP: alkaline phosphatase
ALT: alanine aminotransferase
AST: aspartate aminotransferase
BMI: body mass index
FPG: fasting plasma glucose
GLP‐1: Glucagon‐like peptide 1
HbA1c: Glycated hemoglobin
HDL‐C: high‐density lipoprotein
HOMA: Homeostasis Model Assessment
hsCRP: high‐sensitivity C‐reactive protein
IFG: impaired fasting glucose
IL‐6: interleukin 6
LDL‐C: low‐density lipoprotein
NFκB: nuclear factor kappa B
OGTT: 2 hours oral glucose tolerance test
PEPCK: Phosphoenolpyruvate carboxykinase
PPARγ: peroxisome proliferator‐activated receptor gamma
TBARS: thiobarbituric acid reactive substances
TEAC: trolox equivalent antioxidant capacity
TNF‐α: tumor necrosis factor alpha
WHO: World Health Organization
γGT: gamma glutamyl transferase

## 1 INTRODUCTION

Rapid aging of the population, combined with sedentary habits and inadequate diet, plays an important role in the epidemic of prediabetes and diabetes. Currently, 425 million people live with diabetes and 374 million have prediabetes worldwide (IDF, 2017). People with prediabetes are defined by three specific parameters: (a) altered fasting glycemia, defined by fasting blood glucose between 6.0 and 6.9 mmol/L (100 to 125 mg/dl); (b) impaired glucose tolerance, defined by oral glucose tolerance test (OGTT) between 7.8 and 11.0 mmol/L (140–200 mg/dl), or the combination of both previous conditions; and (c) HbA1c between 5.7% and 6.4% (ADA, 2018).

The pathophysiology of prediabetes is complex and involves the combination of multiple changes in the mechanisms involved in glucose homeostasis. Similar to type 2 diabetes, prediabetes is associated with increased glucose levels, decreased insulin sensitivity, increased inflammatory cytokines, and altered incretin responses (Brannick, Wynn, & Dagogo‐Jack, 2016). Prediabetes usually has no apparent signs or symptoms but may progress to type 2 diabetes with microvascular and macrovascular complications such as retinopathy, microalbuminuria, neuropathy, and cardiovascular disease. For this reason, to prevent or retard the progression to type 2 diabetes in this population is a relevant therapeutic goal. Recommendations include changes in lifestyle, such as regular physical activity combined with a balanced diet and use of antidiabetic drugs (ADA, 2018; Wasserman, Wang, & Brown, 2018). However, changes in lifestyle can be difficult to maintain in the long run, and antidiabetic drugs may be associated with side effects (Roberts, Craig, Adler, McPherson, & Greenhalgh, 2018).

Nutraceuticals may represent a complementary or adjunct alternative to drugs prescribed in the treatment of prediabetes. Eriomin®, a supplement of citrus flavonoids, composed of eriocitrin, hesperidin, and naringin, has been shown to have anti‐inflammatory, antihyperglycemic, and antioxidant properties (Minato et al., 2003; Miyake et al., 2006; Zaidun, Thent, & Latiff, 2018). Experimental mice (C57BL/6 J) supplemented with eriocitrin (140 mg/kg/day) showed an increase of total antioxidant capacity and decreased inflammatory markers (IL‐6, MCP‐1, and hsPCR) in the blood circulating and organs (Ferreira, Spolidorio, Manthey, & Cesar, 2016). Another study showed that hesperetin supplementation promoted improved plasma glucose, insulin, and glycogen levels in STZ‐induced rats (Jayaraman, Subramani, Sheik Abdullah, & Udaiyar, 2018). An in vitro study showed that naringenin improved hyperglycemia by inhibits Toll‐like receptor 2 expression in adipose tissue of high‐fat diet‐fed mice (Yoshida et al., 2013).

Although the antioxidant and anti‐inflammatory activities of citrus flavonoids have been widely recognized (Parhiz, Roohbakhsh, Soltani, Rezaee, & Iranshahi, 2015; Yi, Ma, & Ren, 2017), there is a lack of information about their actions on metabolic disorders related to prediabetes in humans, as well as information about potentially effective doses. Therefore, this study aimed to investigate the efficacy of three increasing doses of Eriomin® (200, 400, and 800 mg/day) on biochemical, metabolic, and inflammatory markers and the potential of Eriomin for reversal of the prediabetic condition.

## 2 METHODS

### 2.1 Individuals

Subjects were recruited through advertisement in the local media (websites, radio, and newspapers), distribution of fliers in the community, and City Health Center and University email lists. Individuals of both sexes, aged 35–60 years, and with prediabetes were considered eligible to participate in the study, because prediabetes is more prevalent in middle‐aged adults, and below 60 years because the confounding factor of aging. Prediabetic subjects were selected according to the Expert Panel of the American Diabetes Association (2018), which include these following features: (a) impaired glucose metabolism, (b) glucose intolerance, and (c) glycated hemoglobin ≥5.7%.

The exclusion criteria were as follows: pregnancy; smoking; history of diabetes mellitus, cardiovascular, hepatic, renal, disease; use of dietary supplements (vitamins, minerals, bioflavonoids, probiotic, symbiotic, or other bioactive compound); continuous use drugs; history of drug or alcohol abuse; and intense physical exercise (more than 10 hr per week).

### 2.2 Sample size

The primary endpoint was serum glucose, and sample size was estimated based on a similar clinical study (Mohammadi et al., 2015). The sample size for the parallel clinical trial was calculated statistically ( $N=\frac{\left(\sigma_{1}^{2}+\sigma_{2}^{2}\right)\;{\left(Z_{\alpha }+Z_{\beta }\right)}^{2}}{{\left(\mu_{2}-\mu_{1}\right)}^{2}}$), with significance level of 5% and 80% power. The minimum sample size was 24 individuals per group and, in anticipation of a 15% dropout rate, 30 individuals were considered per intervention group.

### 2.3 Ethics approval and consent to participate

The procedures performed in this study followed the ethical guidelines of the National Health Council (Res. 466/12) and Declaration of Helsinki (1964). The protocol was cleared by the Ethical Board of the Pharmacy School of UNESP (CAAE: 67610217.6.0000.5426) and registered at ClinicalTrials.gov (NCT03215043). All individuals signed an Informed Consent Form before starting the study.

### 2.4 Trial design

To evaluate the effectiveness of Eriomin®, three distinct doses were selected based on the previous clinical trials performed with hesperidin, which average dose given was around 500 mg, ranging from 146 to 1,000 mg (Demonty et al., 2010; Homayouni, Haidari, Hedayati, Zakerkish, & Ahmadi, 2018; Mohammadi et al., 2015; Morand et al., 2011). A double‐blind, randomized, placebo‐controlled, parallel‐design trial was conducted between November 2017 and February 2018 according to the CONSORT 2010. All recruited individuals (*n* = 120) were randomized (block size) by random computer‐generated numbers. Subjects were allocated into four groups: (a) Placebo: 30 subjects given a daily dose of 400 mg placebo; (b) Eriomin 200 mg: 30 subjects given a daily dose of 200 mg Eriomin; (c) Eriomin 400 mg: 30 subjects given a daily dose of 400 mg Eriomin; (d) Eriomin 800 mg: 30 subjects given a daily dose of 800 mg Eriomin.

The randomization scheme was performed by an independent researcher. Containers and capsules of Eriomin and placebo were identical and were prepared by a pharmacist who did not participate in the study. The container label held only the patient identification number. Thus, both the investigator and the patients were blind from the time of randomization until the analysis was complete.

### 2.5 Supplements preparation

The intervention product was Eriomin®, supplement of citrus flavonoids provided by Ingredients by Nature TM, Montclair, CA. The purity was determined by HPLC and which contains 70% eriocitrin, 5% hesperidin, 4% naringin, and 1% didymin. The placebo, containing 100% microcrystalline corn starch, was formulated by an independent pharmacist, and its appearance was as similar as possible to the active supplement. Subjects were instructed to consume one capsule after dinner with a glass of water during 12 weeks. Supplement and placebo were given to participants every 2 weeks after randomization.

### 2.6 Study procedures

#### 2.6.1 Laboratory analyzes

Overnight fasting blood samples were obtained in the beginning of the first, fourth, eighth, and 12th interventions at the São Lucas Clinical Analyzes Laboratory, Araraquara‐SP, and blood serum was stored at −80°C. Biochemical markers (fasting glucose, glucose tolerance [OGTT], glycated hemoglobin [HbA1c], insulin, total cholesterol, high‐density lipoprotein [HDL‐cholesterol], and triglycerides) were performed by commercial kits (Labtest, Brazil). Low‐density lipoprotein (LDL‐cholesterol) was calculated (Friedwald, Levy, & Friedrickson, 1972). Homeostasis Model Assessment (HOMA‐IR) was calculated, and the cutoff set was at ≥2.71 (Matthews et al., 1985). Metabolic and inflammatory markers (Glucagon‐like peptide 1 [GLP‐1], glucagon, C‐peptide, adiponectin, tumor necrosis factor alpha [TNF‐α], interleukin 6 [IL‐6], and high‐sensitivity C‐reactive protein [hsCRP]) was performed by Luminex Milliplex® (RP3X Scientific, Ribeirao SP, Brazil). Lipid peroxidation was assessed by TBARS assay (Yagi, 1998) and total antioxidant capacity by radical ABTS + assay (Re et al., 1999). Liver and renal markers (aspartate transaminase [AST], alanine transaminase [ALT], alkaline phosphatase [ALP], and gamma‐glutamyl transferase [γGT] and creatinine) were performed by commercial kits (Labtest, Brazil). Serum creatinine is considered a biomarker of chronic kidney disease and acute renal injury, according to the Brazilian Society of Nephrology (2011).

#### 2.6.2 Anthropometry and blood pressure

In the beginning of the first, fourth, eighth, and 12th weeks, it was evaluated the following anthropometric parameters: body weight (kg), muscle mass (kg), fat mass (kg), body fat (%; InBody 720, Biospace, Tokyo, Japan) body mass index (BMI) was calculated by following computation: weight in kg/height in meters squared and waist‐to‐hip ratio was evaluated according to the usual standards (WHO, 2008). Blood pressure was measured with digital monitor (ReliOn, HEM‐741 CRELN, USA).

#### 2.6.3 Dietary parameters

Subjects were instructed to maintain their usual diet and physical activity during the total experimental period. In the beginning of the first, fourth, eighth, and 12th weeks, registered nutritionists have analyzed usual dietary intake by a 3‐day dietary record nonconsecutive, and the analysis of energy and macronutrient and micronutrient intake was performed using the DietBox®, based on the Brazilian Table of Food Composition (Unicamp, 2006).

### 2.7 Compliance and adverse events

Adverse effects were previously defined by the investigators as the presence of any unfavorable and unintended signs on the health and well‐being of individuals, abnormal laboratory findings, symptoms and/or diseases temporarily associated after administration of Eriomin® or placebo. During the intervention, patients were questioned biweekly by nutritionists for the eventual occurrence of nausea, vomiting, diarrhea, or any change in general well‐being or illness. Conformity was assessed by counting unused capsules at each visit. Participants who consumed more than 90% of the provided capsules and completed all evaluations had good compliance and were included in the statistical analysis.

### 2.8 Primary and secondary outcome measures

The primary outcome was serum fasting glucose. The secondary outcome was glucose tolerance (OGTT), HbA1c, insulin, HOMA‐IR, total cholesterol, triglycerides, HDL‐cholesterol, LDL‐cholesterol, ALP, γGT, AST, ALT, GLP‐1, glucagon, C‐peptide, TNF‐α, IL‐6, hsCRP, antioxidant capacity, serum lipid peroxidation, body weight, body mass index (BMI), muscle mass, fat mass, body fat and waist‐to‐hip ratio, systolic and diastolic blood pressure, and intake of macronutrients and micronutrients.

To avoid spurious association or bias between a major outcome with an independent external variable (confounding variable), such as physical activity, diet, anthropometry or lifestyle, each patient was monitored biweekly to ensure no changes on these parameters. Physical activity was assessed individually at each consultation, asking the type and period expended on it. The diet was evaluated by a 3‐day nonconsecutive dietary record, performed 1 week before the biweekly return to the nutritionist. Anthropometric measurements were taken biweekly.

### 2.9 Statistical analysis

Data are presented as mean ± SD. Statistical analysis was performed using SPSS 22 (Statistical Package Social Sciences). One‐way ANOVA was used to identify differences between groups in the baseline period. Two‐way repeated measures ANOVA followed by Sidak post hoc test were apply to compare changes within and between groups over 12 weeks. The significance was *p* ≤ .05.

## 3 RESULTS

### 3.1 Individuals

**Figure 1:**
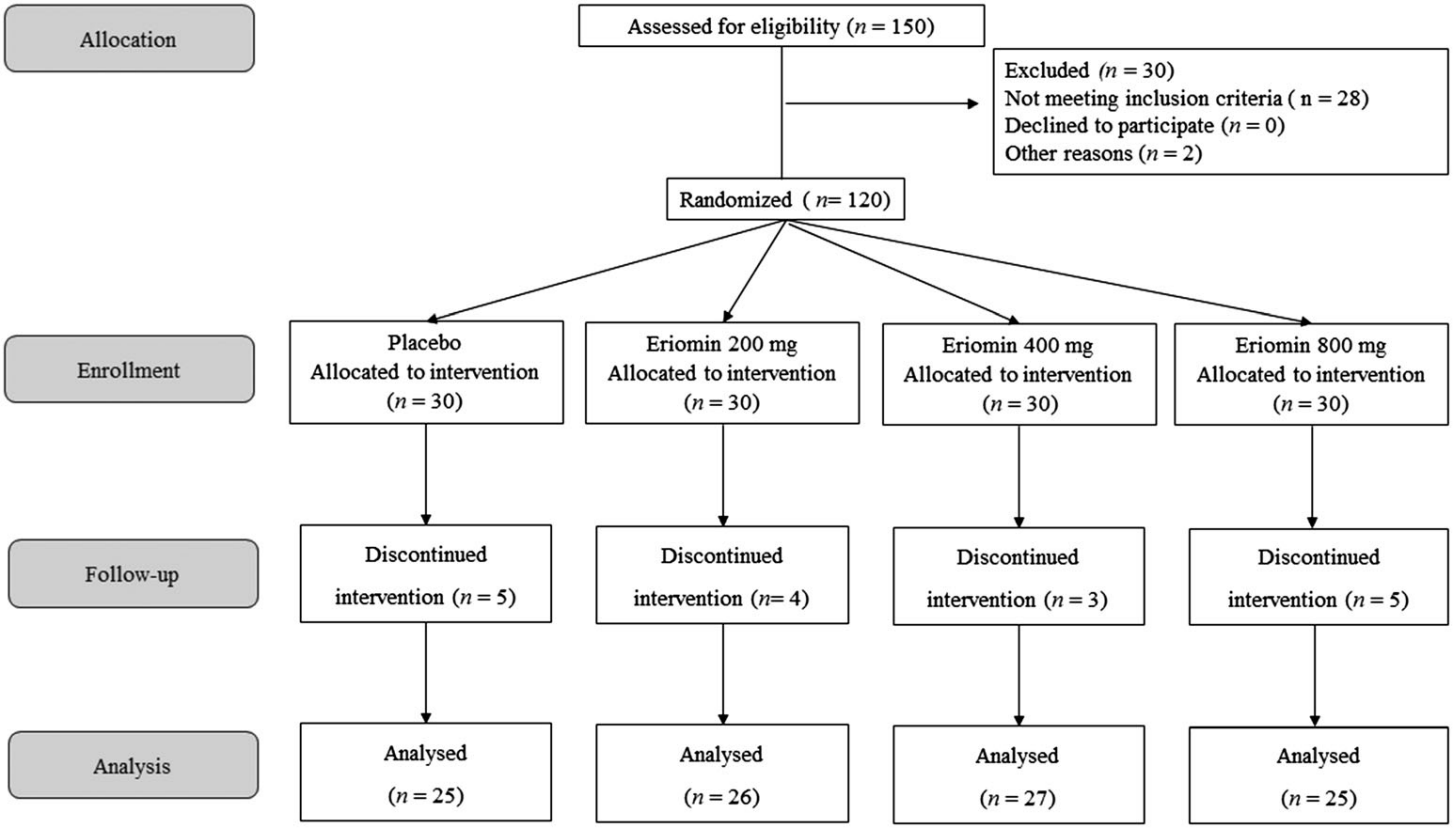
Trial design

One hundred and three subjects, 49 men and 54 women, 49 ± 10 years, previously classified as prediabetic, were included in this study; 17 participants were excluded for the following reasons: low compliance to product intake (i.e., <90% compliance, *n* = 6), disease (*n* = 3), family circumstances (*n* = 2), moved away (*n* = 1), and did not attend samples withdrawal (*n* = 5; Figure 1). The baseline characteristics of all participants were similar among the groups: placebo and 200, 400, and 800 mg Eriomin, as shown in Table 1.

**Table 1:** Baseline characteristics of individuals with prediabetic subject to supplementation with 200, 400, or 800 mg/day for 12 weeks

Variables		Eriomin		
	Placebo	200 mg	400 mg	800 mg
n	25	26	27	25
Age	47.5 ± 12.3	51.3 ± 11.4	48.1 ± 9.8	51.6 ± 8.9
Glucose (mg/dl)	104 ± 10	104 ± 13	104 ± 10	103 ± 13
OGTT (mg/dl)	150 ± 17	149 ± 21	151 ± 19	151 ± 19
Insulin (μU/ml)	19.1 ± 6.5	19.4 ± 7.2	19.3 ± 9.0	19.4 ± 6.6
HOMA‐IR	4.70 ± 2.02	4.70 ± 1.38	4.71 ± 2.26	4.71 ± 1.56
HbA1c (%)	5.81 ± 0.50	5.82 ± 0.42	5.81 ± 0.50	5.80 ± 0.51
Total cholesterol (mg/dl)	193 ± 32	184 ± 41	190 ± 35	184 ± 40
LDL‐cholesterol (mg/dl)	116 ± 19	105 ± 19	112 ± 22	106 ± 35
HDL‐cholesterol (mg/dl)	47.3 ± 11.3	46.3 ± 10.9	46.6 ± 7.91	46.0 ± 10.9
Triglycerides (mg/dl)	146 ± 78	152 ± 41	151 ± 69	162 ± 93
Aspartate transaminase (U/L)	22.1 ± 7.5	22.0 ± 7.0	23.5 ± 7.2	21.5 ± 5.3
Alanine transaminase (U/L)	25.6 ± 15.1	24.1 ± 10.5	27.1 ± 12.9	22.6 ± 7.6
Alkaline phosphatase (U/L)	63.0 ± 15.6	60.8 ± 18.4	59.8 ± 17.6	59.8 ± 17.6
γ Glutamiltransferase (U/L)	42.7 ± 30.8	41.0 ± 34.5	49.8 ± 58.3	34.0 ± 14.4
hsCRP (mg/dl)	0.43 ± 0.36	0.44 ± 0.33	0.42 ± 0.30	0.41 ± 0.42
Glucagon‐like peptide‐1 (pmol/L)	8.48 ± 1.96	8.52 ± 2.72	8.55 ± 2.75	8.57 ± 2.11
Glucagon (pg/ml)	143 ± 19	144 ± 23	142 ± 19	143 ± 14
C‐peptide (pg/ml)	2122 ± 636	2172 ± 727	2163 ± 767	2151 ± 624
IL‐6 (pg/ml)	6.84 ± 4.33	6.83 ± 3.12	6.82 ± 4.43	6.83 ± 4.92
TNF‐α (pg/ml)	5.64 ± 1.73	5.66 ± 1.91	5.67 ± 1.88	5.65 ± 1.79
Lipid peroxidation (MDA; mM)	1.77 ± 0.91	1.77 ± 0.58	1.78 ± 0.67	1.77 ± 0.82
Antioxidant capacity (μM)	1.87 ± 0.03	1.88 ± 0.09	1.88 ± 0.03	1.87 ± 0.09
BMI (kg/m^2^)	34.2 ± 7.5	34.3 ± 7.1	33.9 ± 6.6	34.0 ± 7.0
Ratio waist/hip	1.06 ± 0.15	1.05 ± 0.10	1.06 ± 0.11	1.06 ± 0.17
Systolic blood pressure (mmHg)	135 ± 12	133 ± 10	133 ± 11	134 ± 9
Diastolic blood pressure (mmHg)	78.8 ± 15.6	80.0 ± 8.0	77.8 ± 10.0	81.2 ± 6.7

*Note*. Data are presented as mean ± *SD*. One‐way ANOVA, *p* ≤ .05.

Abbreviations: BMI, body mass index; OGTT, 2‐hr oral glucose tolerance test; HOMA‐IR, homeostasis model assessment–insulin resistance; HbA1c, glycated hemoglobin; hsCRP, high‐sensitivity C‐reactive protein; IL‐6, interleukin 6; TNF‐α, tumor necrosis factor alpha.

### 3.2 Biochemical markers

Fasting blood glucose levels were reduced after Eriomin treatment in all tested doses. The reductions of 6% were observed at a dose of Eriomin of 200 mg (*p* ≤ .01), 5% in 400 mg Eriomin (*p* ≤ .01), and 4% in 800 mg Eriomin (*p* = .041) at the end of treatment. On the other hand, subjects in the placebo group had an increase in fasting blood glucose levels after 12 weeks (Table 2). Glucose of 2 hr after OGTT was significantly reduced by 7% with administration of 200 or 400 mg of Eriomin and 6% with the dose of 800 mg after 12 weeks of treatment (Table 2). In addition, there was a mean reduction of 2% in HbA1c levels in the three groups supplemented with Eriomin (*p* ≤ .05), whereas placebo showed no change during intervention time (Table 2). All volunteers had insulin resistance at the beginning, during, and at the end of the experiment (HOMA‐IR ≥ 2.71), but there was a reduction of 8% after intervention with 200 mg of Eriomin (*p* = .037), 7% with 400 mg (*p* = .043), and 6% with 800 mg (*p* = .042; Table 2). Regarding blood lipids, there was no reduction in total cholesterol, LDL‐cholesterol, HDL‐cholesterol, and triglycerides during and at the end of the experimental period in the placebo group and supplemented with Eriomin (200, 400, and 800 mg; Table 2).

**Table 2:** Biochemical markers of individuals with prediabetic subject to supplementation with 200, 400, or 800 mg/day for 12 weeks

Variables	Period	Eriomin			
	Week	Placebo	200 mg	400 mg	800 mg
Glucose (mg/dl)	0	104 ± 10	104 ± 13_a_	104 ± 10_a_	103 ± 13_a_
	4	104 ± 13_A_	99 ± 11_b, B_	101 ± 12_b, B_	100 ± 12_b, B_
	8	103 ± 14_A_	99 ± 11_b, B_	100 ± 10_b,B_	100 ± 11_b, B_
	12	105 ± 16_A_	98 ± 10_b, B_	99 ± 10_b, B_	99 ± 11_b, B_
	δ _*(12–0 week)*_	*1.0%* a	*−5.8%* a	*−4.8%* a	*−3.9%* a
			*−4.8%* b		
OGGT (mg/dl)	0	150 ± 17	149 ± 21_a_	151 ± 19_a_	151 ± 19_a_
	12	151 ± 22_A_	139 ± 19_b, B_	140 ± 18_b, B_	141 ± 17_b, B_
	δ _*(12–0 week)*_	−*0.7%*	*−6.7%*	*−7.3%*	*−6.6%*
				*−6.9%*	
Insulin (μU/ml)	0	19.1 ± 6.5	19.4 ± 7.2	19.3 ± 9.0	19.4 ± 6.6
	4	19.1 ± 7.6	19.1 ± 7.6	18.9 ± 10.3	19.3 ± 5.8
	8	19.0 ± 8.6	19.1 ± 7.2	18.5 ± 7.6	19.2 ± 8.0
	12	19.0 ± 8.9	18.2 ± 7.1	18.0 ± 8.9	19.2 ± 6.3
	δ _*(12–0 week)*_	*−0.5%*	*−6.2%*	*−6.7%*	*−1.0%*
			*−4.7* a		
HOMA‐IR	0	4.70 ± 2.02	4.70 ± 1.38_a_	4.71 ± 2.26_a_	4.71 ± 1.56_a_
	4	4.70 ± 2.50	4.67 ± 1.95_a_	4.66 ± 2.47_a_	4.64 ± 1.50_a_
	8	4.68 ± 2.83	4.65 ± 1.82_a_	4.58 ± 1.98_a_	4.64 ± 1.64_a_
	12	4.74 ± 2.81_A_	4.31 ± 1.77_b, B_	4.39 ± 1.98_b, B_	4.44 ± 1.69_b, B_
	*δ*_*(12–0 week)*_	*0.9%*	*−8.3%*	*−6.8%*	*−5.7%*
			*−6.9*		
HbA1c (%)	0	5.81 ± 0.50	5.82 ± 0.42_a_	5.81 ± 0.50_a_	5.80 ± 0.51_a_
	4	5.81 ± 0.51	5.80 ± 0.45_a_	5.81 ± 0.52_a_	5.78 ± 0.61_a_
	8	5.80 ± 0.51^A^	5.71 ± 0.45_b, B_	5.72 ± 0.51_b, B_	5.73 ± 0.52_a, A_
	12	5.82 ± 0.53^A^	5.68 ± 0.41_b, B_	5.70 ± 0.42_b, B_	5.70 ± 0.51_b, B_
	*δ*_*(12–0 week)*_	*0.2%*	*−2.4%*	*−1.9%*	*−1.7%*
			*−2.0%*		
Total cholesterol (mg/dl)	0	193 ± 32	184 ± 41	190 ± 35	184 ± 40
	4	193 ± 29	181 ± 38	190 ± 37	184 ± 41
	8	193 ± 24	181 ± 40	188 ± 30	182 ± 40
	12	195 ± 27	181 ± 40	187 ± 36	182 ± 40
	*δ*_*(12–0 week)*_	*1.0%*	*−1.6%*	*−1.6%*	*−1.1%*
			*−1.4%*		
LDL‐cholesterol (mg/dl)	0	116 ± 19	105 ± 19	112 ± 22	106 ± 35
	4	116 ± 17	103 ± 24	112 ± 30	105 ± 37
	8	116 ± 23	103 ± 27	110 ± 23	103 ± 29
	12	117 ± 23	103 ± 26	110 ± 23	103 ± 25
	*δ*_*12–0 week)*_	*0.9%*	*−1.9%*	*−1.8%*	*−2.8%*
			*−2.2%*		
HDL‐cholesterol (mg/dl)	0	47.3 ± 11.3	46.3 ± 10.9	46.6 ± 7.91	46.0 ± 10.9
	4	47.4 ± 10.0	45.5 ± 9.96	45.5 ± 6.81	45.7 ± 9.91
	8	47.4 ± 11.9	45.5 ± 9.36	45.1 ± 8.07	44.1 ± 10.7
	12	47.6 ± 10.7	45.5 ± 10.6	45.1 ± 8.41	44.0 ± 10.7
	*δ*_*(12–0 week)*_	*0.6%*	*−1.7%*	*−3.2%*	*−4.3%*
			*−3.1%*		
Triglycerides (mg/dl)	0	146 ± 78	152 ± 41	151 ± 69	162 ± 93
	4	145 ± 88	158 ± 51	143 ± 60	153 ± 80
	8	148 ± 77	144 ± 61	149 ± 60	152 ± 75
	12	150 ± 81	144 ± 42	144 ± 74	153 ± 65
	*δ*_*(12–0 week)*_	*2.7%*	*−5.3%*	*−4.6%*	*−5.6%*
			*−5.2%*		

*Note*. Two‐way repeated measures ANOVA followed by Sidak test among groups (placebo, 200, 400, and 800 mg) over 12‐week intervention period; *p* ≤ .05. Different letters (a, b) indicate difference within the group, and different uppercase letters (A, B) indicate difference between groups.

a Percentage difference between week 12 and 0.

b Mean of the percentage differences between the groups supplemented with Eriomin.

### 3.3 Metabolic and inflammatory markers

Levels of blood plasma GLP‐1 increased 15% for all tested doses of Eriomin: 200, 400, and 800 mg (*p* < .001). After intervention, Eriomin supplementation also promoted an average reduction of 6.5% of glucagon (*p* < .001) and 5% of C‐peptide levels (*p* < .001). No change was observed in the placebo group during treatment (Table 3).

**Table 3:** Metabolic and inflammatory markers of individuals with prediabetic subject to supplementation with 200, 400, or 800 mg/day for 12 weeks

Variables	Period	Eriomin			
	Week	Placebo	200 mg	400 mg	800 mg
Glucagon (pg/ml)	0	143 ± 19	144 ± 23_a_	142 ± 19_a_	143 ± 14_a_
	12	142 ± 20_A_	134 ± 21_b, B_	135 ± 19_b, B_	132 ± 14_b, B_
	*δ*_*(12–0 week)*_	*−0.7%* a	*−6.9%* a	*−4.9%* a	*−7.7%* a
				*−6.5%* b	
C‐peptide (pg/ml)	0	2122 ± 636	2172 ± 727_a_	2163 ± 767_a_	2151 ± 624_a_
	12	2106 ± 675_A_	2047 ± 613_b, B_	2055 ± 803_b, B_	2046 ± 648_b, B_
	*δ*_*(12–0 week)*_	*−0.8%*	*−5.8%*	*−5.0%*	*−4.9%*
				*−5.2%*	
GLP‐1 (rmol/L)	0	8.48 ± 1.96	8.52 ± 2.72_a_	8.55 ± 2.75_a_	8.57 ± 2.11_a_
	12	8.50 ± 1.96_A_	9.85 ± 2.68_b, B_	9.80 ± 1.95_b, B_	9.89 ± 2.15_b, B_
	*δ*_*(12–0 week)*_	*0.2%*	*15.6%*	*14.6%*	*15.4%*
			*15.2%*		
hsCRP (mg/dl)	0	0.43 ± 0.36	0.44 ± 0.33_a_	0.42 ± 0.30_a_	0.41 ± 0.42_a_
	12	0.43 ± 0.41_A_	0.38 ± 0.25_b, B_	0.39 ± 0.34_b, B_	0.35 ± 0.30_b, B_
	*δ*_*(12–0 week)*_	*0.0%*	*−13.6%*	*−7.1%*	*−14.6%*
			*−11.8%*		
IL‐6 (pg/ml)	0	6.84 ± 4.33	6.83 ± 3.12_a_	6.82 ± 4.43_a_	6.83 ± 4.92_a_
	12	6.82 ± 4.61_A_	5.89 ± 2.23_b, B_	5.84 ± 3.04_b, B_	6.03 ± 3.80_b, B_
	*δ*_*(12–0 week)*_	*−0.3%*	*−13.8%*	*−14.4%*	*−11.7%*
			*−13.3%*		
TNF‐α (pg/ml)	0	5.64 ± 1.73	5.66 ± 1.91_a_	5.67 ± 1.88_a_	5.65 ± 1.79_a_
	12	5.63 ± 2.03_A_	4.98 ± 1.59_b, B_	5.06 ± 1.63_b, B_	5.02 ± 1.96_b, B_
	*δ*_*(12–0 week)*_	*−0.2%*	*−12.0%*	*−10.8%*	*−11.2%*
			*−11.3%*		
Adiponectin (μg/ml)	0	18.4 ± 5.6	18.7 ± 5.7_a_	17.9 ± 7.5_a_	18.1 ± 7.6_a_
	12	18.7 ± 5.3_A_	22.2 ± 6.6_b, B_	21.8 ± 8.9_b, B_	21.1 ± 6.6_b, B_
	*δ*_*(12–0 wk)*_	*1.6%*	*18.7%*	*21.8%*	*16.6%*
				*19.0%*	
Lipid peroxidation (MDA; mM)	0	1.77 ± 0.91	1.77 ± 0.58_a_	1.78 ± 0.67_a_	1.77 ± 0.82_a_
	12	1.76 ± 0.94_A_	1.33 ± 0.67_b, B_	1.53 ± 0.86_b, B_	1.54 ± 0.84_b, B_
	*δ*_*(12–0 week)*_	*−0.6%*	*−24.9%*	*−14.0%*	*−13.0%*
			*−17.3%*		
Antioxidant capacity (μM)	0	1.87 ± 0.03	1.88 ± 0.09_a_	1.88 ± 0.03_a_	1.87 ± 0.09_a_
	12	1.88 ± 0.03_A_	1.99 ± 0.04_b, B_	1.99 ± 0.03_b, B_	1.98 ± 0.03_b, B_
	*δ*_*(12–0 week)*_	*0.5%*	*5.9%*	*5.9%*	*5.3%*
			*5.7%*		

*Note*. Two‐way repeated measures ANOVA followed by Sidak test among groups (placebo, 200, 400, and 800 mg) over 12‐week intervention period; *p* ≤ .05. Different letters (a, b) indicate difference within the group, and different uppercase letters (A, B) indicate difference between groups.

a Difference between week 12 and 0.

b Mean of the percentage differences between the groups supplemented with Eriomin.

Eriomin supplementation for all doses tested (200, 400, and 800 mg/day) promoted a mean reduction of 12% in hsCRP levels (*p* < .050), 13% in IL‐6 (*p* = .034), 12% in TNF‐α (*p* = .041), 17% in lipid peroxidation levels (*p* < .010) after 12 weeks. Serum adiponectin levels increased by 19% in the Eriomin 200 mg group (*p* < 0.010), 22% in 400 mg (*p* < .010), and 17% in 800 mg (*p* < .050) after 12 weeks. In addition, individuals supplemented with Eriomin had a 6% increase in antioxidant capacity (*p* = .031). Placebo showed no change in these parameters during intervention (Table 3).

### 3.4 Liver and renal markers

Hepatic enzymes (AST, ALT, ALP, and γGT) and a marker of renal function (creatinine) remained unchanged during the experiment in all three groups supplemented with Eriomin and at placebo (Table 4).

**Table 4:** Liver and renal markers of individuals with prediabetic subject to supplementation with 200, 400, or 800 mg/day for 12 weeks

Variables	Period	Eriomin			
	week	Placebo	200	400	800
Aspartate transaminase (AST; U/L)	0	22.1 ± 7.5	22.0 ± 7.0	23.5 ± 7.2	21.5 ± 5.3
	4	22.5 ± 7.5	21.5 ± 5.9	23.6 ± 7.4	23.6 ± 6.6
	8	22.3 ± 7.3	23.3 ± 9.0	22.9 ± 7.9	22.8 ± 7.7
	12	23.4 ± 8.4	23.3 ± 7.8	23.4 ± 8.0	21.8 ± 6.3
	δ _*(12–0 week)*_ a	*5.9%* a	*5.9%* a	*−0.4%* a	*1.4%* a
			*2.3%* b		
Alanine transaminase (ALT; U/L)	0	25.6 ± 15.1	24.1 ± 10.5	27.1 ± 12.9	22.6 ± 7.6
	4	25.1 ± 13.1	23.1 ± 8.3	29.8 ± 16.0	24.8 ± 9.2
	8	26.2 ± 15.7	26.6 ± 12.4	27.8 ± 13.7	25.5 ± 12.3
	12	26.0 ± 15.7	25.2 ± 12.5	28.7 ± 15.1	23.5 ± 8.4
	δ _*(12–0 week)*_ a	*1.6%*	*4.6%*	*5.9%*	*4.0%*
			*4.8%*		
Alkaline phosphatase (ALP; U/L)	0	63.0 ± 15.6	60.8 ± 18.4	59.8 ± 17.6	59.8 ± 17.6
	4	62.4 ± 15.6	60.7 ± 21.4	57.3 ± 17.6	57.3 ± 17.6
	8	64.4 ± 17.9	61.8 ± 23.0	61.3 ± 21.9	61.3 ± 21.9
	12	64.8 ± 15.9	62.6 ± 26.1	62.3 ± 20.1	62.3 ± 20.1
	*δ*_*(12–0 week)*_ a	*2.9%*	*3.0%*	*4.8%*	*4.2%*
			*4.0%*		
Gamma‐glutamyl transferase (γGT; U/L)	0	42.7 ± 30.8	41.0 ± 34.5	49.8 ± 58.3	34.0 ± 14.4
	4	41.9 ± 29.4	42.9 ± 38.5	49.8 ± 44.4	35.3 ± 23.4
	8	44.8 ± 32.1	43.0 ± 32.7	50.0 ± 49.5	35.7 ± 20.9
	12	45.4 ± 33.0	41.5 ± 31.5	51.2 ± 49.8	36.3 ± 27.1
	*δ*_*(12–0 week)*_	*6.6%*	*1.2%*	*2.8%*	*6.8%*
			*3.6%*		
Creatinine (mg/dl)	0	0.80 ± 0.18	0.85 ± 0.21	0.82 ± 0.24	0.83 ± 0.16
	4	0.83 ± 0.19	0.84 ± 0.20	0.80 ± 0.18	0.80 ± 0.15
	8	0.82 ± 0.20	0.85 ± 0.16	0.81 ± 0.19	0.81 ± 0.17
	12	0.83 ± 0.22	0.85 ± 0.18	0.81 ± 0.21	0.80 ± 0.18
	*δ*_*(12–0 week)*_	*3.8%*	*0.0%*	*−1.2%*	*−3.6%*
			*−1.6%*		

*Note*. Two‐way repeated measures ANOVA followed by Sidak test among groups (placebo, 200, 400, and 800 mg) over 12‐week intervention period; *p* ≤ .05.

a Percentage difference between week 12 and 0.

b Mean of the percentage differences between the groups supplemented with Eriomin.

### 3.5 Anthropometry and blood pressure

Supplementation with Eriomin (200, 400, and 800 mg/day) and placebo had no effect on body weight, BMI, lean mass, fat mass, fat percentage, and hip waist ratio. However, all doses of Eriomin supplementation promoted a mean systolic blood pressure reduction of 7%. Diastolic blood pressure remained unchanged in the groups throughout the study (Table 5).

**Table 5:** Anthropometry and blood pressure of individuals with prediabetic subject to supplementation with 200, 400, or 800 mg/day for 12 weeks

Variables	Period	Eriomin			
	week	Placebo	200	400	800
Systolic blood pressure (mmHg)	0	135 ± 12	133 ± 10_a_	133 ± 11_a_	134 ± 9_a_
	4	133 ± 12	133 ± 10_a_	133 ± 11_a_	134 ± 9_a_
	8	134 ± 10	128 ± 12_a_	131 ± 11_a_	129 ± 10_a_
	12	134 ± 10_A_	123 ± 11_b, B_	122 ± 13_b, B_	123 ± 9_b, B_
	*δ*_*(12–0 week)*_	*−0.7%* a	*−7.5%* a	*−8.3%* a	*−8.2%* a
			*−8.0%* b		
Diastolic blood pressure (mmHg)	0	78.8 ± 15.6	80.0 ± 8.0	77.8 ± 10.0	81.2 ± 6.7
	4	77.2 ± 11.7	80.0 ± 7.0	77.8 ± 10.1	80.4 ± 6.8
	8	76.4 ± 11.1	80.0 ± 9.8	77.4 ± 9.8	79.2 ± 8.1
	12	76.2 ± 11.8	78.8 ± 9.9	77.4 ± 9.9	78.0 ± 6.5
	*δ*_*(12–0 week)*_	*−3.3%*	*−1.5%*	*−0.5%*	*−3.9%*
			*−2.0%*		
Body weight (kg)	0	95.3 ± 24.5	96.0 ± 22.0	95.9 ± 18.8	96.0 ± 20.9
	4	95.3 ± 24.6	96.0 ± 22.0	95.7 ± 19.1	96.0 ± 21.6
	8	95.2 ± 24.8	96.1 ± 21.7	95.8 ± 19.2	95.7 ± 21.7
	12	95.5 ± 24.3	96.1 ± 22.1	95.7 ± 19.1	95.8 ± 22.0
	*δ*_*(12–0 week)*_	*0.2%*	*0.1%*	*−0.2%*	*−0.2%*
			*−0.1%*		
BMI (kg/m^2^)	0	34.2 ± 7.5	34.3 ± 7.1	33.9 ± 6.6	34.0 ± 7.0
	4	34.4 ± 7.4	34.3 ± 7.1	33.7 ± 6.8	34.0 ± 7.2
	8	34.2 ± 7.6	34.3 ± 7.1	33.8 ± 6.7	33.9 ± 7.2
	12	34.5 ± 6.7	34.2 ± 6.2	33.7 ± 6.9	34.2 ± 7.1
	*δ*_*(12–0 week)*_	*0.9%*	*0.3%*	*−0.6%*	*0.6%*
			*0.3%*		
Lean mass (kg)	0	32.3 ± 6.7	32.1 ± 6.2	31.8 ± 5.5	32.0 ± 6.9
	4	32.2 ± 7.2	32.4 ± 6.1	32.0 ± 5.8	32.0 ± 7.3
	8	31.9 ± 7.2	32.2 ± 6.2	32.0 ± 5.8	31.9 ± 7.3
	12	32.4 ± 7.1	32.4 ± 6.4	32.0 ± 5.6	31.9 ± 7.4
	*δ*_*(12–0 week)*_	*0.3%*	*0.9%*	*0.6%*	*−0.3%*
			*0.4%*		
Fat mass (kg)	0	38.7 ± 15.6	38.5 ± 16.8	37.8 ± 15.4	39.0 ± 14.3
	4	38.8 ± 15.1	38.2 ± 17.0	37.4 ± 15.6	38.8 ± 14.7
	8	38.8 ± 15.1	38.5 ± 16.8	37.3 ± 15.6	38.0 ± 15.2
	12	38.9 ± 15.2	38.2 ± 16.9	37.3 ± 15.5	38.0 ± 15.4
	*δ*_*(12–0 week)*_	*0.5*	*−0.8%*	*−1.3%*	*−2.6%*
			*−1.6%*		
Body fat (%)	0	39.0 ± 9.3	38.9 ± 9.9	38.8 ± 10.7	39.6 ± 8.8
	4	39.1 ± 8.8	38.7 ± 10.2	38.7 ± 11.1	38.9 ± 9.1
	8	39.6 ± 8.4	38.9 ± 10.0	38.5 ± 11.2	38.8 ± 9.5
	12	39.7 ± 8.5	38.5 ± 9.9	38.6 ± 10.8	38.7 ± 9.7
	*δ*_*(12–0 week)*_	*1.8%*	*−1.0%*	*−0.5%*	*−2.3%*
			*−1.3%*		
Ratio waist/hip	0	1.06 ± 0.15	1.05 ± 0.10	1.06 ± 0.11	1.06 ± 0.17
	4	1.06 ± 0.19	1.05 ± 0.10	1.05 ± 0.12	1.05 ± 0.17
	8	1.06 ± 0.19	1.05 ± 0.10	1.05 ± 0.12	1.05 ± 0.18
	12	1.06 ± 0.12	1.05 ± 0.10	1.05 ± 0.10	1.05 ± 0.12
	*δ*_*(12–0 week)*_	*0.0%*	*0.0%*	*−0.9%*	*−0.9%*
			*−0.6%*		

*Note*. Two‐way repeated measures ANOVA followed by Sidak test among groups (placebo, 200, 400, and 800 mg) over 12‐week intervention period; *p* ≤ 0.05 Different letters (a, b) indicate difference within the group, and different uppercase letters (A, B) indicate difference between group.

a Percentage difference between week 12 and 0.

b Mean of the percentage differences between the groups supplemented with Eriomin.

### 3.6 Dietary parameters

Intake of energy and macronutrients (carbohydrates, proteins, and lipids) and cholesterol, saturated fatty acid, fibers, vitamin E, and vitamin C were not significantly altered in patients supplemented with Eriomin or placebo during the 12‐week intervention (Table 6).

**Table 6:** Dietary parameters of individuals with prediabetic subject to supplementation with 200, 400, or 800 mg/day for 12 weeks

Variables	Period	Eriomin			
	Week	Placebo	200	400	800
Energy (Kcal)	0	1820 ± 242	1879 ± 214	1769 ± 174	1840 ± 270
	4	1815 ± 264	1893 ± 223	1771 ± 182	1832 ± 257
	8	1817 ± 240	1881 ± 229	1772 ± 192	1840 ± 260
	12	1829 ± 294	1873 ± 240	1768 ± 184	1845 ± 255
	*δ*_*(12–0 week)*_	*0.7%* †	*−0.3%* †	*−0.05%* †	*0.2%* †
			*−0.18%* ††		
Carbohydrates (g)	0	253 ± 34	255 ± 32	248 ± 25	253 ± 40
	4	251 ± 37	256 ± 34	250 ± 26	250 ± 38
	8	252 ± 34	255 ± 35	249 ± 26	252 ± 37
	12	254 ± 41	254 ± 36	248 ± 26	254 ± 37
	*δ*_*(12–0 week)*_	*0.3%*	*−0.3%*	*0%*	*0.4%*
			*0.2%*		
Protein (g)	0	68.5 ± 9.4	68.8 ± 5.4	68.1 ± 8.9	69.8 ± 9.6
	4	68.0 ± 9.9	69.3 ± 6.0	68.8 ± 9.1	69.7 ± 10.7
	8	68.3 ± 9.3	68.8 ± 6.1	69.0 ± 8.7	69.9 ± 10.2
	12	68.6 ± 11.2	68.7 ± 5.9	68.0 ± 8.8	70.1 ± 10.7
	*δ*_*(12–0 week)*_	*0.1%*	*−0.14%*	*−0.14%*	*0.4%*
			*−0.22%*		
Lipids (g)	0	61.5 ± 8.6	62.6 ± 7.1	61.5 ± 5.5	62.1 ± 8.4
	4	61.0 ± 9.6	63.1 ± 7.5	61.9 ± 5.6	62.3 ± 8.0
	8	61.2 ± 8.5	62.7 ± 7.6	61.7 ± 6.3	62.9 ± 8.5
	12	61.7 ± 10.2	62.2 ± 7.9	61.6 ± 6.4	62.8 ± 8.3
	*δ*_*(12–0 week)*_	*0.3%*	*−0.6%*	*0.16%*	*1.1%*
			*0.62%*		
Cholesterol (mg)	0	244 ± 35	242 ± 28	243 ± 29	246 ± 25
	4	245 ± 38	243 ± 23	246 ± 29	247 ± 23
	8	246 ± 36	247 ± 22	250 ± 24	252 ± 19
	12	244 ± 35	242 ± 28	245 ± 28	248 ± 23
	δ _(12–0 *week*)_	*0%*	*0%*	*0.8%*	*0.8%*
			*0.5%*		
Saturated fatty acid (SFA; g)	0	20.0 ± 3.2	20.2 ± 2.3	20.3 ± 3.0	21.2 ± 2.3
	4	20.5 ± 3.4	20.7 ± 2.5	20.8 ± 2.9	21.7 ± 2.5
	8	21.5 ± 3.4	20.6 ± 2.9	21.8 ± 3.1	22.0 ± 2.5
	12	20.2 ± 3.3	20.0 ± 2.4	20.5 ± 3.0	21.4 ± 2.4
	δ _(12–0 *week*)_	*1%*	*−1%*	*−0.9%*	*−0.9%*
			*−0.9%*		
Fibers (g)	0	19.4 ± 3.7	19.6 ± 3.5	19.5 ± 3.4	19.5 ± 2.1
	4	19.1 ± 3.1	19.4 ± 3.3	19.1 ± 3.5	19.2 ± 3.8
	8	19.8 ± 3.0	19.9 ± 2.5	19.7 ± 2.9	19.1 ± 3.1
	12	19.3 ± 3.0	19.4 ± 2.5	19.4 ± 2.8	19.3 ± 2.5
	δ _(12–0 *week*)_	*−0.5%*	*−1%*	*−0.5%*	*−1%*
			*0.8%*		
Vitamin E (mg)	0	15.8 ± 3.6	15.4 ± 3.6	15.0 ± 3.1	15.5 ± 1.7
	4	15.5 ± 4.4	15.2 ± 3.1	15.1 ± 2.9	15.6 ± 1.8
	8	15.1 ± 4.8	15.0 ± 3.0	15.0 ± 3.0	15.3 ± 1.9
	12	15.6 ± 4.0	15.5 ± 3.0	15.1 ± 2.9	15.4 ± 2.0
	δ _(12–0 *week*)_	*−1.2%*	*0.6%*	*0.6%*	*−0.6%*
			*0.6%*		
Vitamin C (mg)	0	55.9 ± 4.0	56.5 ± 7.6	55.1 ± 7.0	55.3 ± 6.0
	4	56.2 ± 4.9	56.8 ± 7.7	54.9 ± 6.6	55.7 ± 6.0
	8	55.7 ± 5.1	56.9 ± 7.9	54.4 ± 6.7	55.1 ± 6.0
	12	56.0 ± 4.1	56.8 ± 8.0	55.2 ± 6.8	55.9 ± 6.1
	δ _(12–0 *week*)_	*0.1%*	*0.5%*	*0.1%*	*1%*
			*0.5%*		

*Note*. Two‐way repeated measures ANOVA followed by Sidak test among groups (placebo, 200, 400 ,and 800 mg) over 12‐week intervention period; *p* ≤ .05.

† Percentage difference between week 12 and 0.

†† Mean of the percentage differences between the groups supplemented with Eriomin.

### 3.7 Clinical reversal of prediabetic condition

**Figure 2:**
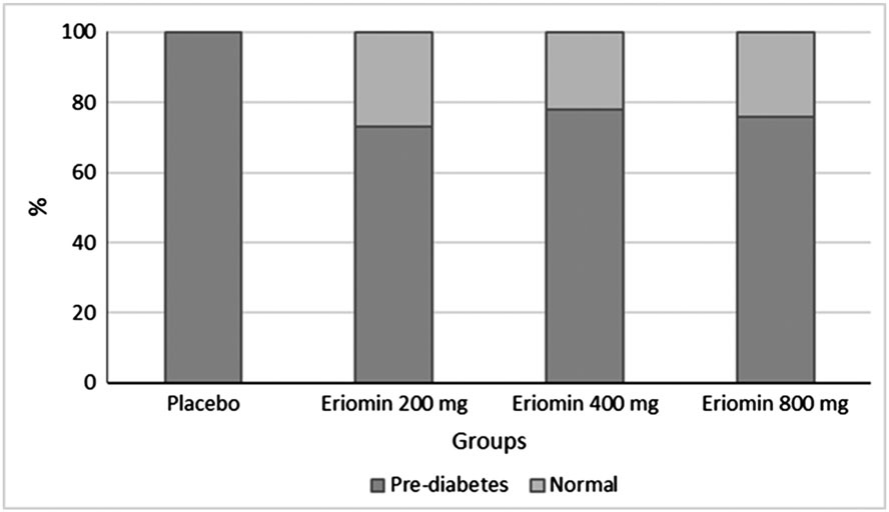
Percentage of individuals with prediabetic after Eriomin supplementation

After 12 weeks, 27% of subjects supplemented with Eriomin 200, 22% with Eriomin 400 mg, and 24% with Eriomin 800 reversed prediabetes to the normal condition (euglycemia). The placebo group maintained the same number of prediabetic individuals (Figure 2).

### 3.8 Adverse effects and safety of Eriomin

During the study, seven adverse events were reported: two cases in the Eriomin 800 group (one pasty stool and one headache), two cases in the 400 group (two pasty stools), one case in the 200 group (one pasty stool), and two cases in the placebo group (one pasty stool and one headache). The frequencies of these events did not differ between groups (*p* > .05), showing that, in general, Eriomin supplementation was well tolerated, without reports of severe or chronic adverse events. Furthermore, liver and kidney functions were unchanged in all groups during treatment.

## 4 DISCUSSION

This study evaluated the efficacy of Eriomin® in the management of hyperglycemia, inflammatory and metabolic parameters, and its contribution to the reversal of the prediabetes state in a short time interval. After 12 weeks of Eriomin supplementation with 200, 400, or 800 mg per day, there was a significant decrease in fasting glycemia, impaired glucose tolerance, HOMA‐IR, HbA1c, glucagon, C‐peptide, hsCRP, IL‐6, and TNF‐α. In addition, there were increases in the blood levels of GLP‐1, adiponectin, and antioxidant capacity. The lack of dose dependence means that there is a biochemical gate regulating the magnitude of the changes measured in this study, saturated at concentrations of 200 mg or less. This means that there may be a set of preliminary biochemical events, very tightly controlled by very low concentrations of Eriomin, for which there is still no clear information.

Under Eriomin supplementation, 5% reduction in glycemic levels and 7% reduction in the glucose tolerance test occurred. Serum HbA1c and insulin resistance, as measured by HOMA‐IR, reduced by 2% and 7%, respectively. These findings were clinically relevant because after treatment with Eriomin 24% of patients reversed the prediabetic clinical condition for normal glycemia and/or lower glucose intolerance. Citrus flavonoids have been identified as antidiabetic compounds because of their hypoglycemic effects, observed in vitro and in vivo studies. (Bucolo, Leggio, Drago, & Salomone, 2012; Fukuchi et al., 2008; Zhang et al., 2012). In type 2 diabetic mice, hesperidin and naringin appear to regulate the activities of hepatic enzymes involved in gluconeogenesis and glycolysis (Jung, Lee, Jeong, & Choi, 2004). These flavanones were also able to reduce the expression of glucose‐6‐phosphatase mRNA, phosphoenolpyruvate carboxykinase (PEPCK), and hepatic GLUT2 and increase GLUT4 expression in adipocytes (Jung, Lee, Park, Kang, & Choi, 2006). Moreover, it was shown that eriodictyol regulates the expression of peroxisome proliferator‐activated receptor gamma (PPARγ) mRNA, in hepatocytes and adipocytes, which activates insulin signaling and promotes translocation of the glucose transporter GLUT4, increasing intracellular glucose uptake and consequently improving insulin sensitivity (Zhang et al., 2012).

It has been reported that prediabetic individuals have impaired serum GLP‐1 secretion and incretin secreted by intestinal L cells after carbohydrate ingestion (Wang et al., 2016). GLP‐1 is involved in regulating glucose metabolism, stimulating insulin secretion, and inhibiting glucagon secretion, thereby lowering plasma glucose levels (Gastaldelli, Gaggini, & DeFronzo, 2017). In our study, all doses of Eriomin promoted a 15% increase in GLP‐1 levels and a 6% reduction in glucagon levels, which presumably may be associated with improved hyperglycemia in prediabetic volunteers. A recent review described new mechanisms of how citrus flavonoids act in the secretion and signaling of GLP‐1 to regulate the glucose metabolism (Domínguez Avila, Rodrigo García, González Aguilar, & Rosa, 2017). This fact was demonstrated in experiments with naringin, a citrus flavanone, which inhibited dipeptidil peptidase 4 (DPP‐4) by increasing the half‐life of GLP‐1, improving insulin secretion and glucose uptake (Parmar et al., 2012).

Another important marker for the prediabetic condition is the low level of adiponectin, an adipokine that plays a crucial role in insulin sensitivity and regulation of glucose metabolism, and is also considered a risk factor for cardiovascular disease (Banerjee et al., 2017; Lai, Lin, Xing, Weng, & Zhang, 2015). Previous studies showed citrus flavonoids positively regulate adiponectin transcription in adipocytes and increase their levels in patients with myocardial infarction (Haidari et al., 2015; Liu et al., 2008). These data corroborate with our findings, where supplementation with Eriomin increased serum adiponectin levels by 18% and reduced the concentration of C‐peptide by 5%, an important marker of beta cell function that allows differentiation between the prognosis of type 1 and type 2 diabetes (Kim et al., 2016).

Increased production of proinflammatory cytokines, such as IL‐6 and TNF‐α, plays an important role in the pathogenesis of type 2 diabetes and contributes to long‐term micro and macrovascular complications (Forbes & Cooper, 2013; Navarro & Mora, 2006). Prediabetic patients exhibit higher levels of these markers during disease progression (Dorcely et al., 2017). Results of our study showed that Eriomin lowered the low‐grade inflammation in prediabetic patients by reductions of serum levels of hsCRP (−12%), IL‐6 (−13%), and TNF‐α (−11%). Previous study in mice supplemented with eriocitrin or eriodictyol also showed decreases in the elevated levels of IL‐6 and hsCRP caused by a high‐fat diet (Ferreira et al., 2016). Another study showed that eriodictyol had inhibitory effects on mRNA expression of IL‐6 and TNF‐α (Lee, 2011). Some mechanisms have been proposed to explain the anti‐inflammatory properties of citrus flavonoids, which include activation of PPARγ expression and inhibition of nuclear factor kappa B (NFκB), with consequent reduction of inflammatory cytokine secretion and increase of adiponectin (Gamo, Miyachi, Nakamura, & Matsuura, 2014; Lee, 2011).

Hyperglycemia is associated with oxidative stress, which plays an important role at the progression of diabetes, insulin resistance, and β‐cell dysfunction (Rehman & Akash, 2017). In contrast, flavonoids act as antioxidants against various diseases by neutralizing the effects oxidative stress (Kawser Hossain et al., 2016). In the present study, Eriomin supplementation resulted in improved blood serum antioxidant status and reduced oxidative stress, evidenced by increased antioxidant capacity (+6%) associated with reduced lipid peroxidation marker (−17%). Our results are consistent with previous studies, which observed a reduction of lipid peroxidation on the blood serum, liver, and kidneys of diabetic rats treated with eriocitrin (Bucolo et al., 2012; Ferreira et al., 2016; Miyake, Yamamoto, Tsujihara, & Osawa, 1998). In addition, eriodictyol protected against kidney injury through activating nuclear factor‐erythroid related factor 2 (Nrf2; Li et al., 2016). In diabetic individuals, the activation of Nrf2 protects pancreatic β cells against various insults, thus maintaining glucose homeostasis and also increasing insulin sensitivity (Sireesh, Dhamodharan, Ezhilarasi, Vijay, & Ramkumar, 2018). The tendency of eriodictyol to inhibit free radical‐mediated events is governed by its chemical structure. Double bonds, hydroxyl groups, and carbonyl function confer antioxidant and anti‐apoptotic properties of flavonoid molecules (Bucolo et al., 2012). According to Miyake et al. (1998), eriocitrin has stronger antioxidant activity than the other citrus flavonoid compounds due to its multiple hydroxyl groups.

Eriomin supplementation during 12 weeks was well tolerated by patients, because AST, ALT, ALP, and γGT levels remained at normal levels. Altered levels of these enzymes reflect damage to hepatocytes and are considered sensitive and specific clinical biomarkers for hepatotoxicity (Ozer, Ratner, Shaw, Bailey, & Schomaker, 2008). These results suggest absence or nondetectable toxicity or impairment of liver function, as previously shown of lemon flavonoid supplementation (Hiramitsu et al., 2014). In addition, a study with mice showed that eriocitrin protected against liver damage caused by consumption of the high‐fat diet (Ferreira et al., 2016).

This study showed strong aspects, including (a) double‐blinded, placebo‐controlled design, (b) high adherence of patients attested by the low number of withdrawal, and (c) evaluation of Eriomin adverse effects and toxicity (presence of any unfavorable and unintended signs, liver enzymes and creatinine). However, some limitations were also observed, such as the relatively short duration of the study (12 weeks), and indirect observation of dietary intake and physical activity through recorded. More studies with longer intervention time and larger sample sizes are needed to better understand the effects of Eriomin in attenuating hyperglycemia in prediabetic subjects. In addition, this lack of dose response suggests that studies with doses below 200 mg should be performed.

In conclusion, this study showed that short‐term intervention with Eriomin® benefited glycemic control, lowered the systemic inflammation and oxidative stress, and reversed the prediabetic condition in 24% of total patients evaluated for all dose tested.

## CONFLICT OF INTEREST

The sponsors had no role in the design, execution, interpretation, or writing of the study.

## AUTHOR CONTRIBUTIONS

Carolina Ribeiro enrolled participants, collected data, and wrote the first version of the paper; Fernanda Ramos enrolled participants and collected data; John Manthey conceived the study and provided chemical analysis. Thais Cesar and Carolina Ribeiro designed the study, and Thais Cesar corrected the first version of the paper. All authors contributed significantly to analysis and interpretation of data, discussion, editing, and approval of the final version of this paper.
